# An explicit form of the polynomial part of a restricted partition function

**DOI:** 10.1007/s40993-016-0065-3

**Published:** 2017-01-05

**Authors:** Karl Dilcher, Christophe Vignat

**Affiliations:** 1 Department of Mathematics and Statistics, Dalhousie University, Halifax, Nova Scotia B3H 4R2 Canada; 2 LSS-Supelec, Université Paris-Sud, Orsay, France; 3 Department of Mathematics, Tulane University, New Orleans, LA 70118 USA

**Keywords:** Restricted partitions, Sylvester waves, Bernoulli polynomials, Raabe’s identity, Primary 11P81, Secondary 11B68

## Abstract

We prove an explicit formula for the polynomial part of a restricted partition function, also known as the first Sylvester wave. This is achieved by way of some identities for higher-order Bernoulli polynomials, one of which is analogous to Raabe’s well-known multiplication formula for the ordinary Bernoulli polynomials. As a consequence of our main result we obtain an asymptotic expression of the first Sylvester wave as the coefficients of the restricted partition grow arbitrarily large.

## Background

An interesting topic in the theory of partitions is that of *restricted partitions*, where the following question has been studied quite extensively: given a vector $\mathbf{d }:=(d_1,d_2,\ldots ,d_m)$ of positive integers, let $W(s,\mathbf{d })$ be the number of partitions of the integer *s* with parts in $\mathbf{d }$, i.e., $W(s,\mathbf{d })$ is the number of solutions of

1 $$\begin{aligned} d_1x_1+d_2x_2+\cdots +d_mx_m = s \end{aligned}$$

in nonnegative integers $x_1,\ldots ,x_m$. For a history of this problem, see [5, p. 119ff.].

A standard method of dealing with questions of this type goes back to Euler and involves a generating function, which in our case is

2 $$\begin{aligned} F(t,{\mathbf{d }}) :=\prod _{j=1}^m\frac{1}{1-t^{d_j}} = \sum _{s=0}^\infty W(s,{\mathbf{d }})t^s. \end{aligned}$$

A major advance was made by Sylvester [22, 23] who wrote the restricted partition function $W(s,{\mathbf{d }})$ as a sum of “waves”,

3 $$\begin{aligned} W(s,{\mathbf{d }}) = \sum _{j\ge 1}W_j(s,{\mathbf{d }}), \end{aligned}$$

where the sum is taken over all distinct divisors *j* of the components of ${\mathbf{d }}$. Sylvester [23] showed that for each such *j*, $W_j(s,{\mathbf{d }})$ is the coefficient of $t^{-1}$, i.e., the residue, of the function

4 $$\begin{aligned} F_j(s,t) = \sum _{\begin{array}{c} 0\le \nu <j\\ \gcd (\nu ,j)=1 \end{array}} \frac{\rho _j^{-\nu s}e^{st}}{\left( 1-\rho _j^{\nu d_{1}}e^{-d_{1}t}\right) \cdots \left( 1-\rho _j^{\nu d_{m}}e^{-d_{m}t}\right) }, \end{aligned}$$

where $\rho _j$ is a primitive *j*th root of unity, for instance $\rho _j=e^{2\pi i/j}$, and where we set $\gcd (0,0)=1$ by convention. In other words, the sum in (4) is taken over all primitive *j*th roots of unity $\rho _j^{\nu }$. These *Sylvester waves* have been studied in great detail in recent years; see, e.g., [10, 19, 20]; see also [7, 8, 14] for a broader perspective, and [21] for computations related to restricted partitions.

For $j=1$, the right-hand side of (4) is recognizable as being very close to the generating function of a higher-order Bernoulli polynomial. This fact was used by Rubinstein and Fel [20] to write $W_1(s,{\mathbf{d }})$ in a very compact form in terms of a single higher-order Bernoulli polynomial [see (33) below]. A version of this result, given in two different forms, was earlier obtained by Beck, Gessel and Komatsu [3], as mentioned in [20]. Similarly, for $j=2$ we have $\rho _j=-1$, and the right-hand side of (4) will typically lead to a convolution sum of higher-order Bernoulli and higher-order Euler polynomials; this was also done in [20]. Furthermore, Rubinstein and Fel extended this approach and expressed $W_j(s,{\mathbf{d }})$ for arbitrary *j* in terms of generalized Eulerian polynomials of higher order, in addition to the expected higher-order Bernoulli polynomials.

In a subsequent paper, Rubinstein [19] showed that all the Sylvester waves $W_j(s,{\mathbf{d }})$ can be written as linear combinations of the first wave ($j=1$) alone, with modified integers *s* and vectors ${\mathbf{d }}$ [see (47) below]. This makes it worthwhile to give further consideration to $W_1(s,{\mathbf{d }})$, which is the purpose of the present paper. Our main result is the following explicit formula for $W_1(s,{\mathbf{d }})$; its significance lies in the fact that it does not contain Bernoulli numbers or polynomials.

### Theorem 1.1

Let ${\mathbf{d }}:=(d_1,d_2,\ldots ,d_m)$ be given, and denote $d:=d_1\ldots d_m$ and  $\widetilde{d}_i:=d/d_i$, $i=1,\ldots ,m$. Then

5 $$\begin{aligned} W_1(s,{\mathbf{d }}) = \frac{1}{(m-1)!d^m} \sum _{\begin{array}{c} 0\le \ell _1\le \widetilde{d}_1-1\\ \dots \\ 0\le \ell _m\le \widetilde{d}_m-1 \end{array}} \prod _{j=1}^{m-1}\left( s+jd-\ell _1d_1-\dots -\ell _md_m\right) . \end{aligned}$$

For a more compact form of this identity, see Sect. 5.

Towards proving this theorem, we derive (or re-derive) some identities which are analogous to classical results in the theory of Bernoulli polynomials and their higher-order analogues. Our main tool is a symbolic notation which, in spite of some similarities, is different from the classical umbral calculus. This will be introduced in Sect. 2, and we apply it in Sect. 3 to prove the auxiliary results as well as Theorem 1.1. In Sect. 4 we present some examples and consequences of Theorem 1.1, including an asymptotic expression. We finish this paper with some additional remarks in Sect. 5.

## Symbolic notation

Although the results in Sect. 3 could also be proved (and in some cases have been proved) by other methods, especially using generating functions, the symbolic notation described below makes the discovery and proof of some identities considerably easier. While there are similarities to the classical umbral calculus (see, e.g., [11] or [18]), our notation is more specific to Bernoulli numbers and polynomials, and is related to probability theory. The following brief exposition is partly taken from [6]; we repeat it here for the sake of completeness.

The basis for our notation are two symbols, ${\mathcal {B}}$ and ${\mathcal {U}}$, which annihilate each other, as we shall see. First, we define the *Bernoulli symbol* ${\mathcal {B}}$ by

6 $$\begin{aligned} {\mathcal {B}}^{n}=B_{n}\qquad (n=0, 1,\ldots ), \end{aligned}$$

where $B_n$ is the *n*th Bernoulli number. So, for instance, we can be rewrite the usual definition for the Bernoulli polynomial $B_n(x)$,

7 $$\begin{aligned} B_n(x)=\sum _{j=0}^n\left( {\begin{array}{c}n\\ j\end{array}}\right) B_jx^{n-j}\quad \hbox {as}\;\; B_n(x) = (x+{\mathcal {B}})^n. \end{aligned}$$

Furthermore, with the usual (generating function) definition of the Bernoulli numbers we have

8 $$\begin{aligned} \exp \left( {\mathcal {B}}z\right) = \sum _{n=0}^\infty {\mathcal {B}}^n\frac{z^n}{n!} = \sum _{n=0}^\infty B_n\frac{z^n}{n!} = \frac{z}{e^z-1}. \end{aligned}$$

We obtain a useful identity from this if we note that

$$\begin{aligned} \exp (({\mathcal {B}}+1)z)=\frac{z}{e^z-1}\cdot e^z = \frac{-z}{e^{-z}-1} =\exp (-{\mathcal {B}}z), \end{aligned}$$

and thus

9 $$\begin{aligned} {\mathcal {B}}+1 = -\mathcal {B}. \end{aligned}$$

We also require several independent Bernoulli symbols ${\mathcal {B}}_{1},\ldots ,{\mathcal {B}}_{k}$. Independence means that if we have any two Bernoulli symbols, say ${\mathcal {B}}_1$ and $\mathcal {B}_2$, then

10 $$\begin{aligned} {\mathcal {B}}_1^{k}\mathcal {B}_2^{\ell }=B_kB_\ell . \end{aligned}$$

Second, the *uniform symbol* ${\mathcal {U}}$ is defined by

11 $$\begin{aligned} f(x+{\mathcal {U}})=\int _0^1f(x+u)du. \end{aligned}$$

Here and elsewhere we assume that *f* is an arbitrary polynomial. From (11) we immediately obtain, in analogy to (6),

12 $$\begin{aligned} {\mathcal {U}}^{n}=\frac{1}{n+1}\qquad (n=0, 1,\ldots ), \end{aligned}$$

and using this, we get

13 $$\begin{aligned} \exp \left( {\mathcal {U}}z\right) =\sum _{n=0}^\infty {\mathcal {U}}^n\frac{z^n}{n!} =\frac{e^z-1}{z}. \end{aligned}$$

From (8) and (13) we now deduce

$$\begin{aligned} \exp \left( z\left( {\mathcal {B}}+{\mathcal {U}}\right) \right) =\sum _{n=0}^\infty \left( {\mathcal {B}}+{\mathcal {U}}\right) ^n\frac{z^n}{n!} = 1, \end{aligned}$$

which means that ${\mathcal {B}}$ and ${\mathcal {U}}$ annihilate each other, i.e., $({\mathcal {B}}+{\mathcal {U}})^n=0$ for all $n\ne 0$, in the sense that

14 $$\begin{aligned} f(x+{\mathcal {B}}+{\mathcal {U}})=f(x), \end{aligned}$$

for an arbitrary polynomial *f*.

For an integer $m\ge 1$ and a collection of not necessarily distinct real numbers $\{a_1,\ldots ,a_m\}$ we now introduce the *discrete uniform symbol* ${\mathcal {U}}_{\{a_1,\ldots ,a_m\}}$ by way of the generating function

15 $$\begin{aligned} \exp \left( z{\mathcal {U}}_{\{a_1,\ldots ,a_m\}}\right) = \frac{e^{a_1z}+\dots +e^{a_mz}}{m}, \end{aligned}$$

or equivalently by

16 $$\begin{aligned} f\left( x+{\mathcal {U}}_{\{a_1,\ldots ,a_m\}}\right) = \frac{1}{m}\bigl (f(x+a_1)+\cdots +f(x+a_m)\bigr ), \end{aligned}$$

for an arbitrary polynomial *f*, which can be seen as a discrete analogue of (11). From the definition (15) we immediately obtain the identity

17 $$\begin{aligned} c\,{\mathcal {U}}_{\{a_1,\ldots ,a_m\}} ={\mathcal {U}}_{\{ca_1,\ldots ,ca_m\}} \qquad (c\in {\mathbb R}). \end{aligned}$$

Furthermore, given two sets ${\mathbf{a }}=\{a_1,\ldots ,a_m\}$ and ${\mathbf{b }}=\{b_1,\ldots ,b_n\}$, we have

$$\begin{aligned} \exp (z({\mathcal {U}}_{{\mathbf{a }}}+{\mathcal {U}}_{\mathbf{b }}))&= \frac{1}{m}\left( \sum _{i=1}^m e^{a_iz}\right) \frac{1}{n}\left( \sum _{j=1}^n e^{b_jz}\right) = \frac{1}{mn}\sum _{\begin{array}{c} 1\le i\le m\\ 1\le j\le n \end{array}}e^{(a_i+b_j)z} \\&= \exp (z{\mathcal {U}}_{\{a_1+b_1,\ldots ,a_m+b_n\}}), \end{aligned}$$

and thus

18 $$\begin{aligned} {\mathcal {U}}_{\{a_1,\ldots ,a_m\}}+{\mathcal {U}}_{\{b_1,\ldots ,b_n\}} ={\mathcal {U}}_{\{a_1+b_1,\ldots ,a_m+b_n\}}, \end{aligned}$$

with an obvious extension (by induction) to an arbitrary number of summands.

Considering the special case $\{0,1,\ldots ,m-1\}$, we multiply (15) and (13) and get

$$\begin{aligned} \exp \left( z({\mathcal {U}}+U_{\{0,1,\ldots ,m-1\}})\right) = \frac{e^z-1}{z}\cdot \frac{1+e^z+\dots +e^{(m-1)z}}{m} = \frac{e^{mz}-1}{mz}, \end{aligned}$$

and thus, using again (13),

19 $$\begin{aligned} m{{\mathcal {U}}} = \mathcal {U} + U_{\{0,1,\ldots ,m-1\}}. \end{aligned}$$

But by (14) we have ${\mathcal {B}}+{\mathcal {U}}=0$ and $m({\mathcal {B}}+{\mathcal {U}})=0$, so that by (19) we have ${\mathcal {U}}+m{\mathcal {B}}+{\mathcal {U}}_{\{0,1,\ldots ,m-1\}}=0$. Since ${\mathcal {B}}+{\mathcal {U}}=0$, we deduce

20 $$\begin{aligned} {\mathcal {B}} = m\mathcal {B} + U_{\{0,1,\ldots ,m-1\}}. \end{aligned}$$

Finally, for an integer $k\ge 1$ we define the *higher-order Bernoulli symbol* ${\mathcal {B}}^{(k)}$ by

21 $$\begin{aligned} {\mathcal {B}}^{(k)} = \mathcal {B}_1+\cdots +{\mathcal {B}}_k, \end{aligned}$$

where ${\mathcal {B}}_1,\ldots ,\mathcal {B}_k$ are independent Bernoulli symbols; see (10).

## Higher-order Bernoulli polynomials and proof of Theorem 1.1

One of the most remarkable and useful identities for the classical Bernoulli polynomials is Raabe’s formula [16] of 1851,

22 $$\begin{aligned} B_n(mx) = m^{n-1}\sum _{j=0}^{m-1}B_n\left( x+\tfrac{j}{m}\right) , \end{aligned}$$

valid for all integers $m\ge 1$ and $n\ge 0$; see also [15, (24.4.18)].

For an integer $k\ge 1$, the *Bernoulli polynomial of order* *k* is defined by the generating function

23 $$\begin{aligned} \left( \frac{z}{e^z-1}\right) ^ke^{xz} = \sum _{n=0}^{\infty }B_n^{(k)}(x)\frac{z^n}{n!}. \end{aligned}$$

The following identity can be seen as a higher-order analogue of Raabe’s formula.

### Theorem 3.1

Let *n*, *m*, and $d_1,\ldots ,d_m$ be positive integers, and set $d:=d_1\ldots d_m$. Then

24 $$\begin{aligned} \left( x+d_1{\mathcal {B}}_1+\ldots +d_m{\mathcal {B}}_m\right) ^n =d^{n-m+1}\sum _{\ell } B_n^{(m)}(\tfrac{x}{d}+\ell ), \end{aligned}$$

where the sum is taken over all values

$$\begin{aligned} \ell =\frac{1}{d}(\ell _1d_1+\cdots +\ell _md_m), \quad 0\le \ell _i\le \frac{d}{d_i}-1,\quad i=1,\ldots ,m. \end{aligned}$$

### Proof

Let $\widetilde{d}_i:=d/d_i$ for $1\le i\le m$. Then using (20) with ${\mathcal {B}}$ replaced by ${\mathcal {B}}_i$ and *m* by $\widetilde{d}_i$, we get

$$\begin{aligned} \sum _{i=1}^m d_i{\mathcal {B}}_i = \sum _{i=1}^m d_i\widetilde{d}_i{\mathcal {B}}_i +\sum _{i=1}^m d_i{\mathcal {U}}_{\{0,1,\ldots ,\widetilde{d}_i-1\}} =d{\mathcal {B}}^{(m)}+\sum _{i=1}^m d_i{\mathcal {U}}_{\{0,1,\ldots ,\widetilde{d}_i-1\}}, \end{aligned}$$

where we have used (21) and the fact that, by definition, $d_i\widetilde{d}_i=d$ for all $i=1,\ldots ,m$. Thus,

25 $$\begin{aligned} \left( x+d_1{\mathcal {B}}_1+\cdots +d_m{\mathcal {B}}_m\right) ^n =d^n\left( \frac{x}{d}+{\mathcal {B}}^{(m)} +\frac{1}{d}\sum _{i=1}^m d_i{\mathcal {U}}_{\{0,1,\ldots ,\widetilde{d}_i-1\}}\right) ^n. \end{aligned}$$

Now, using (17), followed by an iterated version of (18), we get

$$\begin{aligned} \frac{1}{d}\sum _{i=1}^m d_i{\mathcal {U}}_{\{0,1,\ldots ,\widetilde{d}_i-1\}} =\sum _{i=1}^m\frac{1}{\widetilde{d}_i}{\mathcal {U}}_{\{0,1,\ldots ,\widetilde{d}_i-1\}} =\sum _{i=1}^m{\mathcal {U}}_{\{0,\tfrac{1}{\widetilde{d}_i},\ldots ,\tfrac{\widetilde{d}_i-1}{\widetilde{d}_i}\}} = {\mathcal {U}}_{\{\ell \}}, \end{aligned}$$

where $\{\ell \}$ indicates the collection of all values

$$\begin{aligned} \ell = \frac{\ell _1}{\widetilde{d}_1}+\cdots +\frac{\ell _m}{\widetilde{d}_m}\quad 0\le \ell _i\le \widetilde{d}_i-1,\quad i=1,\ldots ,m. \end{aligned}$$

Thus we have with (25),

26 $$\begin{aligned} \left( x+d_1{\mathcal {B}}_1+\cdots +d_m{\mathcal {B}}_m\right) ^n =d^n\left( \frac{x}{d}+{\mathcal {B}}^{(m)}+{\mathcal {U}}_{\{\ell \}}\right) ^n. \end{aligned}$$

Finally we note that the number of (not necessarily distinct) elements in $\{\ell \}$ is

27 $$\begin{aligned} \widetilde{d}_1\ldots \widetilde{d}_m = \frac{d^m}{d_1\ldots d_m} = d^{m-1}. \end{aligned}$$

Therefore by (16), in this case with $d^{m-1}$ in place of *m*, (26) leads to (24), and we are done. $\square$

We note that this result is not new. In fact, it is a special case of identity (60) in the classical book of Nörlund [13, p. 135]. However, the method of proof in [13] is very different from ours and relies on the theory of finite differences. On the other hand, it should also be mentioned that the symbolic notation involving the Bernoulli symbol, more or less as used on the left-hand side of (24), can also be found in [13], on p. 135 and elsewhere.

Among the numerous known results about higher-order Bernoulli polynomials which can be found, for instance, in [13, Ch. 6], the identity

28 $$\begin{aligned} B_{m-1}^{(m)}(x) = (x-1)(x-2)\dots (x-m+1)\qquad (m\ge 2), \end{aligned}$$

with $B_0^{(1)}(x)=B_0(x)=1$, is of particular importance here; for a proof see, e.g., [13, p. 147].

There is a more general concept of a higher-order Bernoulli polynomial, which allowed Rubinstein and Fel [20] to express the first Sylvester wave in a very compact form. It can be defined as follows (see, e.g., [9, p. 39] or [20, p. 333]): For a fixed $m\ge 1$ and a vector of positive integers ${\mathbf{d }}=(d_1,\ldots ,d_m)$ we define the polynomials $B_n^{(m)}(x|{\mathbf{d }})$, $n=0,1,\ldots$, by the generating function

29 $$\begin{aligned} e^{xz}\prod _{i=1}^m\frac{d_iz}{e^{d_iz}-1} = \sum _{n=0}^\infty B_n^{(m)}(x|{\mathbf{d }})\frac{z^n}{n!}, \end{aligned}$$

or symbolically by

30 $$\begin{aligned} B_n^{(m)}(x|{\mathbf{d }}) =\left( x+d_1{\mathcal {B}}_1+\cdots +d_m{\mathcal {B}}_m\right) ^n. \end{aligned}$$

Comparing (29) with (23), we see that $B_n^{(m)}(x|(1,\ldots ,1))=B_n^{(m)}(x)$. The polynomials $B_n^{(m)}(x|{\mathbf{d }})$, with a different notation and different normalization, can also be found in [2] and [4, p. 151], where they are called *Bernoulli-Barnes polynomials*.

From (24) and (28) we can now obtain the following analogue of (28).

### Corollary 3.2

Let $m\ge 1$ be an integer and ${\mathbf{d }}:=(d_1,\ldots ,d_m)$ a vector of positive integers, and denote $d:=d_1\ldots d_m$ and $\widetilde{d}_i:=d/d_i$ for $1\le i\le k$. Then

31 $$\begin{aligned} B_{m-1}^{(m)}(x|{\mathbf{d }}) = \frac{1}{d^{m-1}} \sum _{\begin{array}{c} 0\le \ell _1\le \widetilde{d}_1-1\\ \cdots \\ 0\le \ell _m\le \widetilde{d}_m-1 \end{array}} \prod _{j=1}^{m-1}\left( x-jd+\ell _1d_1+\dots +\ell _md_m\right) . \end{aligned}$$

Before proving this, we note that in the case $d_1=\cdots =d_m=1$, the multiple sum on the right of (31) collapses to the single term $\ell _1=\dots =\ell _m=0$, and (31) reduces to (28).

### Proof of Corollary 3.2

Using (30) and Theorem 3.1 with $n=m-1$, followed by (28), we get

$$\begin{aligned} B_{m-1}^{(m)}(x|{\mathbf{d }})&= \sum _y B_{m-1}^{(m)}(\tfrac{x}{d}+y) \\&=\sum _{\begin{array}{c} 0\le \ell _1\le \widetilde{d}_1-1\\ \dots \\ 0\le \ell _m\le \widetilde{d}_m-1 \end{array}} \prod _{j=1}^{m-1} \left( \tfrac{x}{d}-j+\tfrac{\ell _1}{\widetilde{d}_1}+\cdots +\tfrac{\ell _m}{\widetilde{d}_m}\right) . \end{aligned}$$

Multiplying each factor in the product on the right by *d*, we obtain (31). $\square$

For the proof of Theorem 1.1 we also need the following reflection formula, which can be found in [13, p. 134]. For the sake of completeness we will provide a proof.

### Lemma 3.3

Let *m* and $d_1,\ldots ,d_m$ be positive integers, and let ${\mathbf{d }}:=(d_1,\ldots ,d_m)$ and $\sigma :=d_1+\cdots +d_m$. Then for all $n\ge 0$ we have

32 $$\begin{aligned} B_n^{(m)}(x+\sigma |{\mathbf{d }}) = (-1)^nB_n^{(m)}(-x|{\mathbf{d }}). \end{aligned}$$

### Proof

Using the definition of $\sigma$ and then (8), we get

$$\begin{aligned} B_n^{(m)}(x+\sigma |{\mathbf{d }})&=\left( x+d_1({\mathcal {B}}_1+1)+\cdots +d_m({\mathcal {B}}_m+1)\right) ^n \\&=\left( x-d_1{\mathcal {B}}_1-\cdots -d_m{\mathcal {B}}_m\right) ^n \\&=(-1)^n\left( -x+d_1{\mathcal {B}}_1+\cdots +d_m{\mathcal {B}}_m\right) ^n; \end{aligned}$$

this last line is the right-hand side of (32). $\square$

We are now ready to prove Theorem 1.1. The previously mentioned identity of Rubinstein and Fel (Eq. (8) in [20]) for the first Sylvester wave is, in our notation,

33 $$\begin{aligned} W_1(s,{\mathbf{d }}) = \frac{1}{(m-1)!d}B_{m-1}^{(m)}(s+\sigma |{\mathbf{d }}), \end{aligned}$$

where, as before, ${\mathbf{d }}=(d_1,\ldots ,d_m)$, $d=d_1\ldots d_m$, and $\sigma =d_1+\dots +d_m$. (A version of (33) can also be found in [4, p. 151].) Now we use (32) with $n=m-1$, followed by (31). This immediately gives (5), and the proof is complete.

## Examples and consequences of Theorem 1.1

**Fig. 1:**
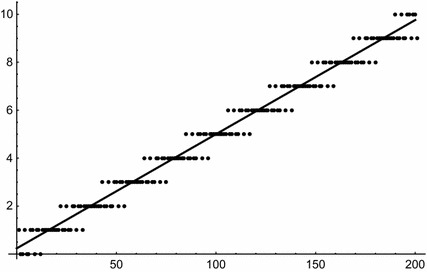
$W_1(s,{\mathbf{d }})$ (*solid line*) and numbers of solutions of (1),i.e., $W(s,{\mathbf{d }})$ (*dots*) for ${\mathbf{d }}=(3,5)$ and $s\le 200$

We begin this section by explicitly stating some small cases of Theorem 1.1 as examples. For $m=2$ we obtain

34 $$\begin{aligned} W_1(s,(d_1,d_2)) = \frac{1}{d_1d_2}\,s+\frac{d_1+d_2}{2d_1d_2}; \end{aligned}$$

this is illustrated by Fig. 1, for ${\mathbf{d }}=(3,5)$.

**Fig. 2:**
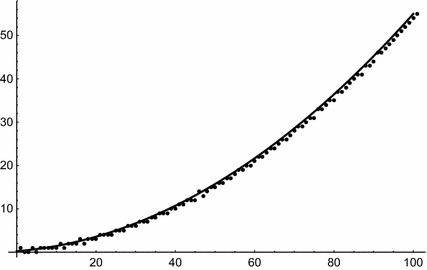
$W_1(s,{\mathbf{d }})$ (*solid line*) and numbers of solutions of (1), i.e., $W(s,{\mathbf{d }})$ (*dots*) for ${\mathbf{d }}=(3,5,7)$ and $s\le 100$

Next, for $m=3$ we have

35 $$\begin{aligned} W_1(s,(d_1,d_2,d_3))&= \frac{1}{2d_1d_2d_3}\,s^2 +\frac{d_1+d_2+d_3}{2d_1d_2d_3}\,s \nonumber \\&\quad \,+\frac{1}{12}\left( \frac{(d_1+d_2+d_3)^2}{d_1d_2d_3}+\frac{1}{d_1}+\frac{1}{d_2}+\frac{1}{d_3}\right) , \end{aligned}$$

which is illustrated by Fig. 2, for ${\mathbf{d }}=(3,5,7)$.

The evaluations (34) and (35) are not new; they can be found in [12, p. 275], where all cases up to $m=7$ are given explicitly. The polynomials (34) and (35), along with the case $m=4$, can also be found in [4, p. 152]. As specific examples, we state below the first few cases for ${\mathbf{d }}=(1,2,\ldots ,m)$, including the specializations of (34) and (35).

36 $$\begin{aligned} W_1(s,(1,2))&= \frac{1}{2}\,s+\frac{3}{4},\end{aligned}$$

37 $$\begin{aligned} W_1(s,(1,2,3))&= \frac{1}{12}\,s^2+\frac{1}{2}\,s+\frac{47}{12},\end{aligned}$$

38 $$\begin{aligned} W_1(s,(1,\ldots ,4))&= \frac{1}{144}\,s^3+\frac{5}{48}\,s^2+\frac{15}{32}\,s +\frac{175}{288},\end{aligned}$$

39 $$\begin{aligned} W_1(s,(1,\ldots ,5))&= \frac{1}{2880}\,s^4+\frac{1}{96}\,s^3+\frac{31}{288}\,s^2 +\frac{85}{192}\,s+\frac{50651}{86400}. \end{aligned}$$

These polynomials also appear in [21, p. 641] as polynomial parts of the identities (2)–(5). Some historical notes with further references can also be found in [21].

Theorem 1.1 can also be used to determine the two highest coefficients of $W_1(s,{\mathbf{d }})$; we state this as a corollary.

### Corollary 4.1

For positive integers $m\ge 2$ and $d_1,\ldots d_m$, we let ${\mathbf{d }}:=(d_1,\ldots d_m)$, $d:=d_1\dots d_m$, and $\sigma :=d_1+\cdots +d_m$, as before. Then

40 $$\begin{aligned} W_1(s,{\mathbf{d }}) =\frac{1}{(m-1)!d}\,s^{m-1}+\frac{\sigma }{2(m-2)!d}\,s^{m-2}+\cdots \end{aligned}$$

The leading coefficient, which has long been known (see, e.g., [3] and references therein), follows immediately from (5) if we note that by (27) the number of summands in the *m*-fold sum is $d^{m-1}$. The second coefficient follows from a simple expansion of the product on the right of (5). We skip the details since both coefficients follow immediately from the identity (3) in [3].

For the special case ${\mathbf{d }}=(1,2,\ldots ,m)$, the identity (40) can be found in [17, Satz 1] and [24, p. 311].

From the fact that the first Sylvester wave is a polynomial, it is clear that for fixed *m* and bounded components of ${\mathbf{d }}$, $W_1(s,{\mathbf{d }})$ is asymptotically equal to the leading term in (40), as *s* gets arbitrarily large. However, it is not immediately clear what happens if the components of ${\mathbf{d }}$ also grow, along with *s*. This is addressed in the following consequence of Theorem 1.1.

### Corollary 4.2

Let $m\ge 2$ be fixed, and consider the vector ${\mathbf{d }}:=(d_1,\ldots ,d_m)$ of positive integers, with $d:=d_1\ldots d_m$. Let $\lambda >0$ and $s\ge \lambda d$, and let *d* grow arbitrarily large in such a way that at least two of the components $d_j$, $1\le j\le m$, are unbounded. Then

41 $$\begin{aligned} W_1(s,{\mathbf{d }}) \sim \frac{1}{(m-1)!d}\,s^{m-1}, \end{aligned}$$

that is, $W_1(s,{\mathbf{d }})$ has the same asymptotic behaviour as in the case of bounded *d*.

The proof of this result relies on interpreting the *m*-fold sum on the right of (5) as a Riemann sum of a certain multiple integral. We therefore begin by evaluating this integral.

### Lemma 4.3

Let $\lambda \in \mathbb R$ be a constant and $m\ge 1$ an integer. Then

42 $$\begin{aligned} \int _{[0,1]^m}\prod _{j=1}^{m-1}(\lambda +j-x_1-\cdots -x_m)dx_1\ldots dx_m =\lambda ^{m-1}. \end{aligned}$$

### Proof

For $m=1$ the product is empty and is therefore 1 by convention; the identity is then trivially true. For $m\ge 2$ we use (28) to rewrite the integral as

$$\begin{aligned} (-1)^{m-1}\int _{[0,1]^m}B_{m-1}^{(m)}(x_1+\cdots +x_m-\lambda )dx_1\ldots dx_m. \end{aligned}$$

Now by (11) each integration over [0, 1] is equivalent to adding a uniform symbol ${\mathcal {U}}$, and since

$$\begin{aligned} B_{m-1}^{(m)}(x_1+\cdots +x_m-\lambda ) = \left( -\lambda +{\mathcal {B}}_1+\cdots +\mathcal {B}_m\right) ^{m-1} \end{aligned}$$

[see, e.g., (30) with ${\mathbf{d }}=(1,\ldots ,1)$], the desired integral is

$$\begin{aligned} (-1)^{m-1}\left( -\lambda +{\mathcal {B}}_1+\cdots +\mathcal {B}_m +{\mathcal {U}}_1+\cdots +\mathcal {U}_m\right) ^{m-1} = \lambda ^{m-1}, \end{aligned}$$

where we have made repeated (*m*-fold) use of the cancellation property (14). $\square$

### Proof of Corollary 4.2

Since $s^{m-1}$ is the highest power in (40), we may as well take $s=\lambda d$. We can then rewrite (5) as

43 $$\begin{aligned} W_1(\lambda d,{\mathbf{d }}) = \frac{1}{(m-1)!d} \sum _{\ell }\prod _{j=1}^{m-1} \left( \lambda +j-\frac{\ell _1}{\widetilde{d}_1}-\cdots -\frac{\ell _m}{\widetilde{d}_m}\right) , \end{aligned}$$

where $\ell$ indicates the summation as detailed in (5). We now denote

$$\begin{aligned} x_j:=\frac{\ell _j}{\widetilde{d}_j}\quad \hbox {and}\quad \Delta x_j = \frac{1}{\widetilde{d}_j} = \frac{d_j}{d},\quad 1\le j\le m. \end{aligned}$$

If only one of the $d_j$, say $d_1$, were unbounded as their product *d* grows, then $\Delta x_1=1/(d_2\ldots d_m)$ would not approach 0 as *d* grows. However, this cannot happen if at least two of the $d_j$ are unbounded as *d* grows. If we now multiply the sum on the right of (43) by

$$\begin{aligned} \Delta x_1\ldots \Delta x_m = \frac{d_1\ldots d_m}{d^m} = \frac{1}{d^{m-1}}, \end{aligned}$$

we can identify this *m*-fold sum as a Riemann sum that converges to the integral in (42). Hence we have, by Lemma 4.3,

$$\begin{aligned} W_1(\lambda d,{\mathbf{d }}) \sim \frac{d^{m-1}}{(m-1)!d}\,\lambda ^{m-1} \quad \hbox {as}\quad d\rightarrow \infty . \end{aligned}$$

Finally, we are done if we replace $\lambda$ by *s* / *d*. $\square$

## Additional Remarks

1. If we divide each factor in the product on the right of (5) by *d*, we see that the resulting product can be written as a Pochhammer symbol (rising factorial) or as a falling factorial. But we can also combine it with $(m-1)!$ in the denominator; using the (generalized) binomial coefficient $$\left( {\begin{array}{c}x\\ n\end{array}}\right) =x(x-1)\ldots (x-n+1)/n!$$ we can then rewrite Theorem 1.1 as follows.

### Corollary 5.1

Let ${\mathbf{d }}:=(d_1,d_2,\ldots ,d_m)$ and $d:=d_1\ldots d_m$. Then

44 $$\begin{aligned} W_1(s,{\mathbf{d }}) = \frac{1}{d} \sum _{\ell }\left( {\begin{array}{c}m-1+\tfrac{s-\ell }{d}\\ m-1\end{array}}\right) , \end{aligned}$$

where the sum is taken over all $\ell$ with

$$\begin{aligned} \ell = \ell _1d_1+\cdots +\ell _md_m,\quad 0\le \ell _i\le \tfrac{d}{d_i}-1,\quad i=1,\ldots ,m. \end{aligned}$$

The binomial coefficient on the right of (44) is reminiscent of some combinatorial objects related to partitions and compositions. (Note, however, that $(s-\ell )/d$ is generally not an integer).

If we set $d_1=\dots =d_m=1$, then the sum in (44) collapses to a single term, as does the sum in (3), and we get

$$\begin{aligned} W(s,{\mathbf{d }}) = W_1(s,{\mathbf{d }}) = \left( {\begin{array}{c}m-1+s\\ m-1\end{array}}\right) . \end{aligned}$$

This is a well-known elementary expression for the number of solutions of (2) for ${\mathbf{d }}=(1,\ldots ,1)$; see, e.g., [2, p. 1328].

While this is not the same as the number of compositions of *s* into *m* parts, there is a connection: The number of compositions of *n* into exactly *m* parts, each at least *k*, is $$\left( {\begin{array}{c}m-1+n-km\\ m-1\end{array}}\right) $$; see [1, p. 63].

2. For the sake of completeness we cite the main result of Rubinstein [19], which we referred to in the Introduction. While for $W_1(s,{\mathbf{d }})$ the order of the components in the given vector ${\mathbf{s }}=(d_1,\ldots ,d_m)$ is irrelevant, this becomes an issue for the Sylvester waves $W_j(s,{\mathbf{d }})$ when $j\ge 2$.

Given an integer $j\ge 2$, we now assume that the components in ${\mathbf{d }}$ are sorted in such a way that *j* divides the first $k_j$ components. We denote

$$\begin{aligned} {\mathbf{d }}_j:= (d_1,\ldots ,d_{k_j},jd_{k_j+1},\ldots ,jd_m), \end{aligned}$$

so that all the components in the vector ${\mathbf{d }}_j$ are divisible by *j*.

We also need the *prime radical circulator* $\psi _j(s)$, which for positive integers *s* is defined by

45 $$\begin{aligned} \psi _j(s) := \sum _{\begin{array}{c} 0\le \nu <j\\ \gcd (\nu ,j)=1 \end{array}}\rho _j^s, \end{aligned}$$

where, as before, $\rho _j$ is a primitive *j*th root of unity. For prime *j* we have

46 $$\begin{aligned} \psi _j(s)={\left\{ \begin{array}{ll} \varphi (j), &{} s\equiv 0\pmod {j},\\ \mu (j), &{} s\not \equiv 0\pmod {j} \end{array}\right. } \qquad (j\;\hbox {prime}), \end{aligned}$$

where $\varphi (j)$ and $\mu (j)$ are Euler’s totient function and the Möbius function, respectively. There is also an explicit formula for composite *j*; see [19] and the references therein for further details.

With these notations, Rubinstein’s identity (19) in [19] can be stated as follows, in a slightly different form:

47 $$\begin{aligned} W_j(s,{\mathbf{d }})&= \sum _{\begin{array}{c} 0\le r_{k_j+1}\le j-1\\ \dots \\ 0\le r_m\le j-1 \end{array}} W_1(s-r_{k_j+1}d_{k_j+1}-\cdots -r_md_m,{\mathbf{d }}_j) \nonumber \\&\quad \,\times \psi _j(s-r_{k_j+1}d_{k_j+1}-\cdots -r_md_m). \end{aligned}$$

We conclude this section with two examples, one of which is general, and the second one is more specific.

### Example 1

If *j* does not divide any of the components of ${\mathbf{d }}$, then $k_j=m$, and $W_j(s,{\mathbf{d }}) = 0$ as the sum on the right of (47) is empty. This is consistent with (3) and the statement following it.

### Example 2

Let ${\mathbf{d }}=(2,4,5)$. Then $k_2=2$, and ${\mathbf{d }}_2=(2,4,10)$. By (45) or (46) we have $\psi _2(s)=(-1)^s$. The identity (47) then gives

$$\begin{aligned} W_2(s,{\mathbf{d }})&= \sum _{r_3=0}^1 W_1(s-r_3d_3,{\mathbf{d }}_3)\psi _2(s-r_3d_3) \\&= W_1(s,(2,4,10))\cdot (-1)^s+W_1(s-5,(2,4,10))\cdot (-1)^{s-5} \\&= (-1)^s\bigl (W_1(s,(2,4,10)) - W_1(s-5,(2,4,10))\bigr ). \end{aligned}$$

The two Sylvester waves $W_1$ above can easily be given explicitly by way of (35). The last line above also illustrates the fact that $W_2(s,{\mathbf{d }})$ is a quasipolynomial; see, e.g., [3] or [4, p. 47].
